# Colon metastasis from lung adenocarcinoma with BRAF V600E mutation: A case report

**DOI:** 10.3389/fimmu.2022.970879

**Published:** 2022-08-08

**Authors:** Yuhao Luo, Kelin Mou, Jianmei Wang, Jing Luo, Lin Peng, Hua Ye, Sheng Lin

**Affiliations:** ^1^ Department of Oncology, The Affiliated Hospital of Southwest Medical University, Luzhou, China; ^2^ Nuclear Medicine and Molecular Imaging Key Laboratory of Sichuan Province, Luzhou, China; ^3^ Department of Pathology, The Affiliated Hospital of Southwest Medical University, Luzhou, China; ^4^ Department of Cardiology, The Affiliated Hospital of Southwest Medical University, Luzhou, China; ^5^ Department of Bone and Joint, The Affiliated Hospital of Southwest Medical University, Luzhou, China

**Keywords:** immunotherapy, pd-1, nsclc, braf v600e, colon metastasis

## Abstract

Symptomatic colon metastasis from primary lung cancer is rare in clinical practice. We report the case of a 58-year-old patient with advanced lung adenocarcinoma who developed abdominal symptoms, including abdominal distention and difficulty defecating, after immunotherapy and chemotherapy. The patient was diagnosed with lung adenocarcinoma, and systemic positron emission tomography-computed tomography confirmed multiple lymph node, pleural, and adrenal metastases. Molecular detection indicated BRAF V600E mutation and high programmed death-ligand 1 (PD-L1) expression. After first-line anti-programmed cell death protein 1 immunotherapy combined with chemotherapy, the nodes in the chest remarkably diminished. However, it was followed by colon obstruction, incomplete ileus, and bone metastasis. Endoscopic histological examination confirmed adenocarcinoma but could not identify primary or secondary tumors due to insufficient tissue. We performed colon resection to remove the obstruction, and postoperative tissue pathological microscopy confirmed metastasis from the lung adenocarcinoma. We corroborated the BRAF V600E mutation and high PD-L1 expression and supported the molecular features of lung adenocarcinoma. During hospitalization, the patient presented with unbearable pain in the bone metastases, and palliative radiotherapy was administered. Then, the patient received dabrafenib plus trametinib as the second-line therapy. This report discusses the clinical characteristics, pathology, imaging, molecular profile assessments, and treatment of primary lung adenocarcinoma with colon metastasis.

## Introduction

Non-small cell lung cancer (NSCLC) accounts for approximately 80% of all lung cancer cases, and nearly half of patients with NSCLC have distant metastases at first diagnosis. The most common metastases sites are the brain, bone, liver, adrenal glands, and lungs (1). Colonic metastases are extremely rare. According to previous reports, the postmortem detection rate of gastrointestinal metastasis from NSCLC ranges from 4.6% to 12.2%. However, the incidence rate of colon metastasis with clinical symptoms is approximately 0.1%, and most of these metastases come from lung squamous cell carcinoma (2–9). Colon metastases are associated with poor clinical outcomes. With the recent development of targeted drugs, tumor molecular profile assessment guides appropriate targeted therapies and significantly improves the overall survival of patients with advanced metastatic lung cancer. Somatic activating BRAF V600E mutations have been found in 1–2% of patients with lung adenocarcinoma. BRAF inhibitors, in combination with MEK inhibitors, have become effective molecular targeting agents for patients with BRAF-mutant lung cancer (10). Here, we report a rare case of a patient with a BRAF V600E mutation who presented with colon metastases of primary lung adenocarcinoma and intestinal symptoms.

## Case Description

**Figure 1:**
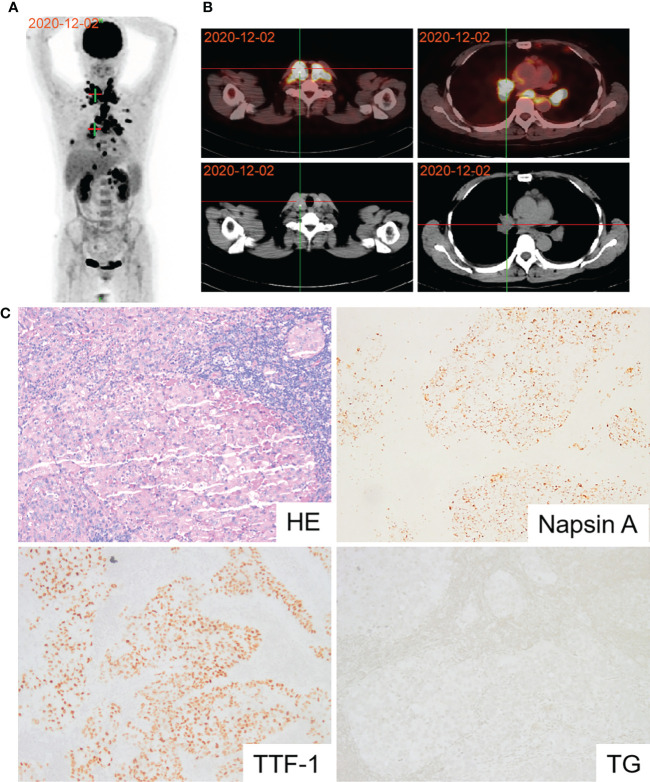
Diagnosis of lung adenocarcinoma using PET and pathology. **(A)** PET-CT scan revealing active metabolic activity in the right lower lobe of the lung, multiple lymph nodes, adrenal gland, pleura and thyroid. **(B)** PET-CT image of thyroid and lung lesions. **(C)** Pathological image of lymph node biopsy confirming lung adenocarcinoma.

A 58-year-old woman presented with symptoms of dyspnea, cough, and chest tightness in December 2020. Echocardiography revealed that the patient’s pericardium contained a large amount of effusion. Pericardiocentesis was performed, and exfoliative cytology was considered malignant. A positron emission tomography-computed tomography (PET-CT) scan showed active metabolic activity in the right lower lobe of the lung, multiple lymph nodes, adrenal gland, pleura, and thyroid per the cancer manifestation (**Figures 1A, B**). A biopsy of the right cervical lymph node was then performed for further diagnosis. Immunohistochemical results of the tissue specimen were positive for CK, CK7, CK19, TTF-1, and napsin A (**Figure 1C**) and negative for CK20, TG, AFP, HepPar1, GPC-3, GATA-3, CD56, CgA, and Syn, which supported the diagnosis of metastasis from lung adenocarcinoma. As TTF-1 is also a marker of thyroid cancer marker (11), and PET-CT also showed metabolic activity in the thyroid gland. We performed a thyroid puncture and excluded the possibility of thyroid cancer. Further molecular detection suggested no alterations in the EGFR, ALK, RET, MET, ROS-1, ERBB2, KRAS and NTRK oncogenes. However, we detected the BRAF V600E mutation and high programmed death-ligand 1 (PD-L1) expression (tumor proportion score ≥ 50%) (**Figure 3A**).

The patient refused targeted therapy, and due to high PD-L1 expression, we administered carrelizumab and pemetrexed plus carboplatin dual drug chemotherapy for six cycles. It was followed by maintenance with carrelizumab plus pemetrexed for six cycles. During immunochemotherapy, chest enhanced CT showed that the perihilar lesions were partially responsive according to RECIST 1.1 (12).

**Figure 2:**
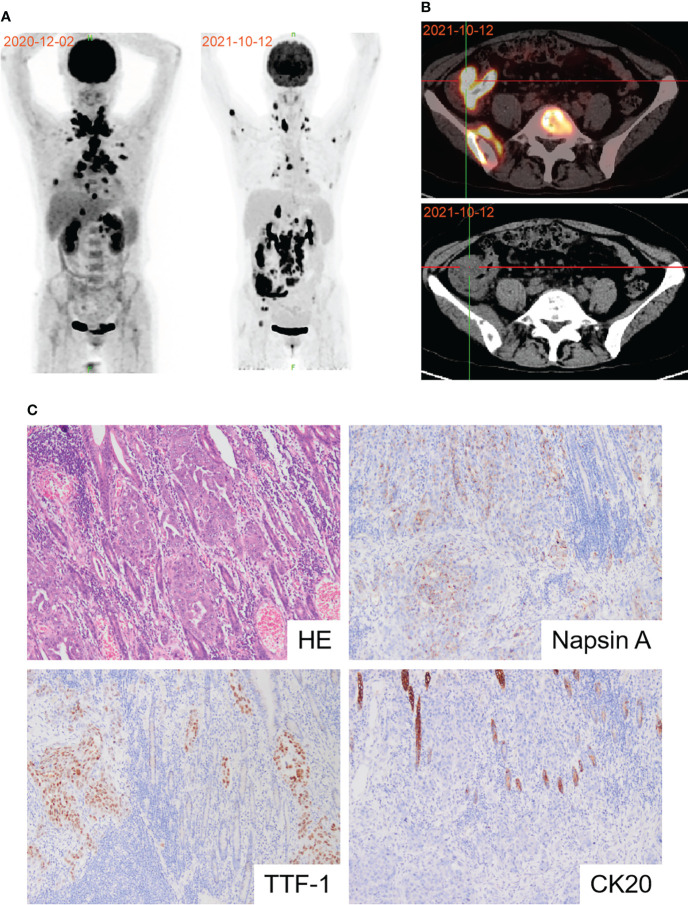
Diagnosis of colon metastasis from lung adenocarcinoma. **(A)** PET-CT scan showed the effects of first-line immunochemotherapy. **(B)** Representative PET-CT image of colon metastasis. **(C)** Pathological image of postoperative colon mass notarized colon metastasis of lung adenocarcinoma.

**Figure 3:**
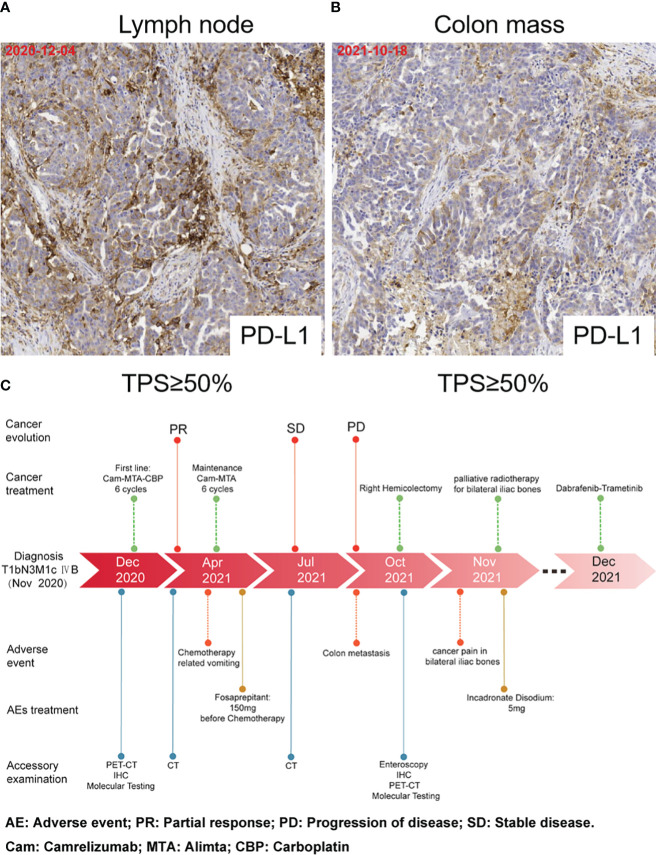
PD-L1 expression and timeline. **(A)** PD-L1 expression in lymph node biopsy tissues. **(B)** PD-L1 expression in the postoperative colon mass specimen. **(C)** Timeline of accessory examination、adverse events and their management during treatment.

The patient was readmitted with symptoms including difficulty defecating, abdominal distension, nausea, and vomiting in October 2021. A new PET-CT scan revealed that the malignant neoplastic lesions in the thorax reduced remarkably. However, the lesions in the distal ileum, ileocecum, and ascending colon occurred concomitant with bone metastases (**Figures 2A, B**). Endoscopic histological examination confirmed adenocarcinoma. However, we could not perform immunohistochemical assessments due to insufficient tissues. Because of the symptoms of refractory intestinal obstruction, the patient underwent a palliative right hemicolectomy and a gastrojejunostomy for abdominal malignancy. Subsequent histopathology revealed that ileocecal and colonic masses were moderately differentiated adenocarcinomas that exhibited positive immunoreactivities for CK7, napsin A, TTF-1, and Villin but were negative for CK20 (**Figure 2C**). Immunohistochemistry of the patient’s postoperative colon mass specimen supported colon metastasis from the lung adenocarcinoma. As colon metastasis from lung cancer is very rare, we speculated whether the secondary colon tumor had the same molecular features as the primary tumor. Further molecular testing suggested that BRAF V600E mutation was also present in the postoperative specimen, and PD-L1 was also highly expressed during testing (**Figure 3B**). Based on the above results, the patient was diagnosed with lung adenocarcinoma with colon, multiple lymph node, and multiple bone metastases.

Our patient had an excellent postoperative recovery but experienced excruciating pain due to the bone metastases in her lower back and lower limbs during hospitalization. Subsequently, the patient was treated with palliative radiotherapy for sacral and bilateral iliac metastases. Based on the BRAF V600E mutation, she received dabrafenib plus trametinib as second-line therapy (**Figure 3C**).

## Discussion

Lung cancer is one of the most common primary malignancies with non-small cell lung cancer accounting for approximately 80% of all lung cancer cases. According to the tissue structure, it can be divided into adenocarcinoma, squamous cell carcinoma, and large cell carcinoma. Approximately 40% of new patients with NSCLC have distant metastases at initial diagnosis (13). The most common sites of NSCLC are the brain (47%), bone (36%), liver (22%), adrenal glands (15%), and lung (11%) (1). According to previous reports, the incidence of gastrointestinal metastasis from lung cancer ranges from 4.6% to 12.2% in postmortem studies (2–5, 7, 9), which is common. However, symptomatic colon metastasis is very rare in clinical studies, with an incidence of approximately 0.1% (8), and lung squamous cell carcinoma is more prone to colon metastasis than lung adenocarcinoma.

The most common clinical presentation of colon metastases in lung cancer patients is abdominal pain due to intestinal obstruction and constipation, which is consistent with the abdominal symptoms in our patient (6). In addition, colon metastases can cause other abdominal symptoms such as diarrhea, bloody stool, intestinal perforation, polyps, and intussusception. Patients with lung cancer often present with colorectal masses on colonoscopy or abdominal CT after the onset of these symptoms. The rarity of colorectal metastasis also poses challenges for the early diagnosis and treatment of patients with advanced lung cancer.

A histological examination can efficiently distinguish between primary and secondary colon cancer. In immunohistochemical staining, CK7 and CK20 are widely used to distinguish tumors of different origins. Primary lung cancer is usually positive for CK7 and negative for CK20 expression. The immune profile of primary colon cancer is positive for CK20 and negative for CK7 (14). In addition, specific markers of lung and colon tumor origin, such as TTF-1 and CDX2, can play roles in the differential diagnosis. Approximately 80% of lung adenocarcinomas express TTF-1 and napsin A, and approximately 90% express at least one of the two markers. Most importantly, TTF-1 and napsin A have almost no co-occurrence in non-pulmonary adenocarcinoma cases (15–18).

Moreover, CDX2, a homeobox gene involved in intestinal differentiation, is expressed in adenocarcinomas of the gastrointestinal tract and other organs. Therefore, specific markers such as TTF-1, napsin A, CDX2, CK7, and CK20 can be used to differentiate between colon metastasis and primary colon cancer. In our case, colon mass immunohistochemistry was positive for CK7, napsin A, and TTF-1 and negative for CK20, supporting the diagnosis of colonic metastasis of primary lung adenocarcinoma.

Most patients with lung cancer do not have colon metastases until after the onset of abdominal symptoms, resulting in a worse 5-year survival rate. Therefore, early diagnosis of metastasis is of great clinical significance for surgery and palliative treatment, which can effectively improve quality of life and shorten the length of hospital stay (6). Histological examination remains the gold standard for tumor diagnosis. Colonoscopy is sufficient to detect colon metastases in patients with primary lung adenocarcinoma presenting abdominal symptoms. Whole-body PET-CT, which is more sensitive than CT, can diagnose distant occult metastasis in patients with lung cancer. PET-CT imaging characteristics combined with tumor markers detection contributes to the diagnosis of benign and malignant lesions, tumor staging, post-treatment efficacy, and enhancing the accuracy of detecting metastases after NSCLC treatment, reducing the likelihood of delayed diagnosis or misdiagnosis. A study of 95 patients with NSCLC confirmed that the sensitivity, accuracy, specificity, positive predictive value, and negative predictive value of a PET-CT were higher than those of the serum carcinoembryonic antigen test (19). Therefore, regular reviews of PET-CTs and continuous monitoring of tumor markers are necessary for patients with primary lung cancer for early assessment and treatment. However, the role of PET-CT in gastrointestinal metastasis of primary lung cancer needs to be assessed in more cases.

It is worth mentioning that we identified a BRAF V600E mutation and high PD-L1 expression in both primary lung cancer tissue and metastatic colon tissue in our patient. BRAF is a serine/threonine kinase downstream of KRAS in the Ras-RAF-MEK-ERK signaling pathway (20). BRAF phosphorylates MEK and promotes tumor cell growth, proliferation, and survival when activated. In clinical studies, BRAF mutations are divided into V600E and non-V600E and occur mainly in melanoma, metastatic colorectal cancer, and papillary thyroid cancer (21–23). The incidence of BRAF mutations in NSCLC is relatively low and ranges from 1% to 3% (24). A study of the clinical characteristics of patients with NSCLC who had BRAF mutations showed that non-smokers predominated in the V600E mutation subgroup (25). No association was observed between sex, age, histology, tumor stage, or performance status with BRAF mutation type. However, most patients with BRAF mutations are diagnosed at an advanced stage and have metastatic disease at the initial diagnosis (26). Per PET-CT and tissue biopsy, our patient was found to have multiple systemic malignant lesions, including metastatic colon carcinoma and papillary thyroid carcinoma. This finding may indicate that patients with NSCLC who have BRAF mutations require a more comprehensive assessment of metastases in the clinic.

Several previous studies have observed that patients with NSCLC who have BRAF mutations may have shorter disease-free, progression-free, and overall survival (27–30). However, recent studies have shown that patients with BRAF V600E mutations have a better prognosis (31). This discrepancy may be related to the fact that the currently available BRAF kinase inhibitors only act on functional mutations of p-V600E. BRAF inhibitors combined with MEK inhibitors, are more effective than monotherapy for treating patients with BRAF mutations (32). Notably, BRAF mutation in NSCLC is associated with high levels of PD-L1 expression.

In contrast to other driver oncogenes, BRAF mutation has no negative impact on susceptibility to immunotherapy (31, 33). However, the combination or choice of BRAF/MEK inhibitors and immunotherapy has not yet been determined. The effectiveness and safety of anti-BRAF-targeted therapy in BRAF-mutated lung adenocarcinoma with colon metastasis are still unclear in clinical practice owing to the limited number of cases. Therefore, large-scale clinical exploration of multiple treatment strategies is necessary. To our knowledge, this is the first reported case of BRAF V600E mutation in primary lung adenocarcinoma with colon metastasis.

Our patient received periodic chemotherapy and immunotherapy with carrelizumab plus pemetrexed after the first molecular detection of BRAF V600E mutation and high PD-L1 expression. The treatment result was significant, and the burdens of the primary tumor and cervical lymph node metastases were generally reduced. However, colon and bone metastases developed. Therefore, dabrafenib plus trametinib was chosen as second-line therapy.

BRAF V600E mutations in colon metastasis from primary lung adenocarcinoma is extremely rare. Pathological immunohistochemistry and molecular testing can assist in distinguishing between colon metastasis and primary colon cancer. According to previous case reports, early detection and surgical intervention can reduce hospital stay, improve quality of life, and improve survival in patients with colon metastasis from primary lung adenocarcinoma. More studies are needed to clarify the efficacy and risks of targeted therapy and immunotherapy in patients with advanced cancer who have BRAF V600E mutations.

## Data availability statement

The original contributions presented in the study are included in the article/**Supplementary Material**. Further inquiries can be directed to the corresponding authors.

## Ethics statement

Written informed consent was obtained from the individual(s) for the publication of any potentially identifiable images or data included in this article.

## Author contributions

YL and SL conceptualized the work. YL and KM drafted the manuscript. JW provided the pathology slides. JL, LP and HY provided the images for publication. All authors contributed to the article and approved the submitted version.

## Funding

This study was supported by the National Natural Science Foundation of China (Nos. 81903000), the Basic Research Foundation of Luzhou People’s Government - Southwest Medical University Plan (Nos. 2019LZXNYDJ22), the Open Project Program of Nuclear Medicine and Molecular Imaging Key Laboratory of Sichuan Province (Nos. HYX19012).

## Acknowledgments

We gratefully acknowledge the patient and her family for their trusting and letting us report this clinical case.

## Conflict of interest

The authors declare that the research was conducted in the absence of any commercial or financial relationships that could be construed as a potential conflict of interest.

## Publisher’s note

All claims expressed in this article are solely those of the authors and do not necessarily represent those of their affiliated organizations, or those of the publisher, the editors and the reviewers. Any product that may be evaluated in this article, or claim that may be made by its manufacturer, is not guaranteed or endorsed by the publisher.

## Supplementary material

Supplementary Figure 1 Imaging results in the period between diagnosis and metastasis(red arrow: lung adenocarcinoma). **(A)** CT image before Camrelizumab+Alimta+Carboplatin. **(B)** CT image after Camrelizumab+Alimta+Carboplatin. **(C)** CT image after Camrelizumab+Alimta.

Supplementary Figure 2 Laboratory results in the period between diagnosis and metastasis. **(A)** Graph showing the dynamics of CEA tumor marker response during treatment. **(B)** Graph showing the dynamics of CA125 tumor marker response during treatment. **(C)** Graph showing the dynamics of CA153 tumor marker response during treatment.
