# Supplementary figures and images for: Colon metastasis from lung adenocarcinoma with BRAF V600E mutation: A case report
Source: Front Immunol. 2022 Aug 8;13:970879. doi: 10.3389/fimmu.2022.970879 (PMC9393296; doi:10.3389/fimmu.2022.970879)

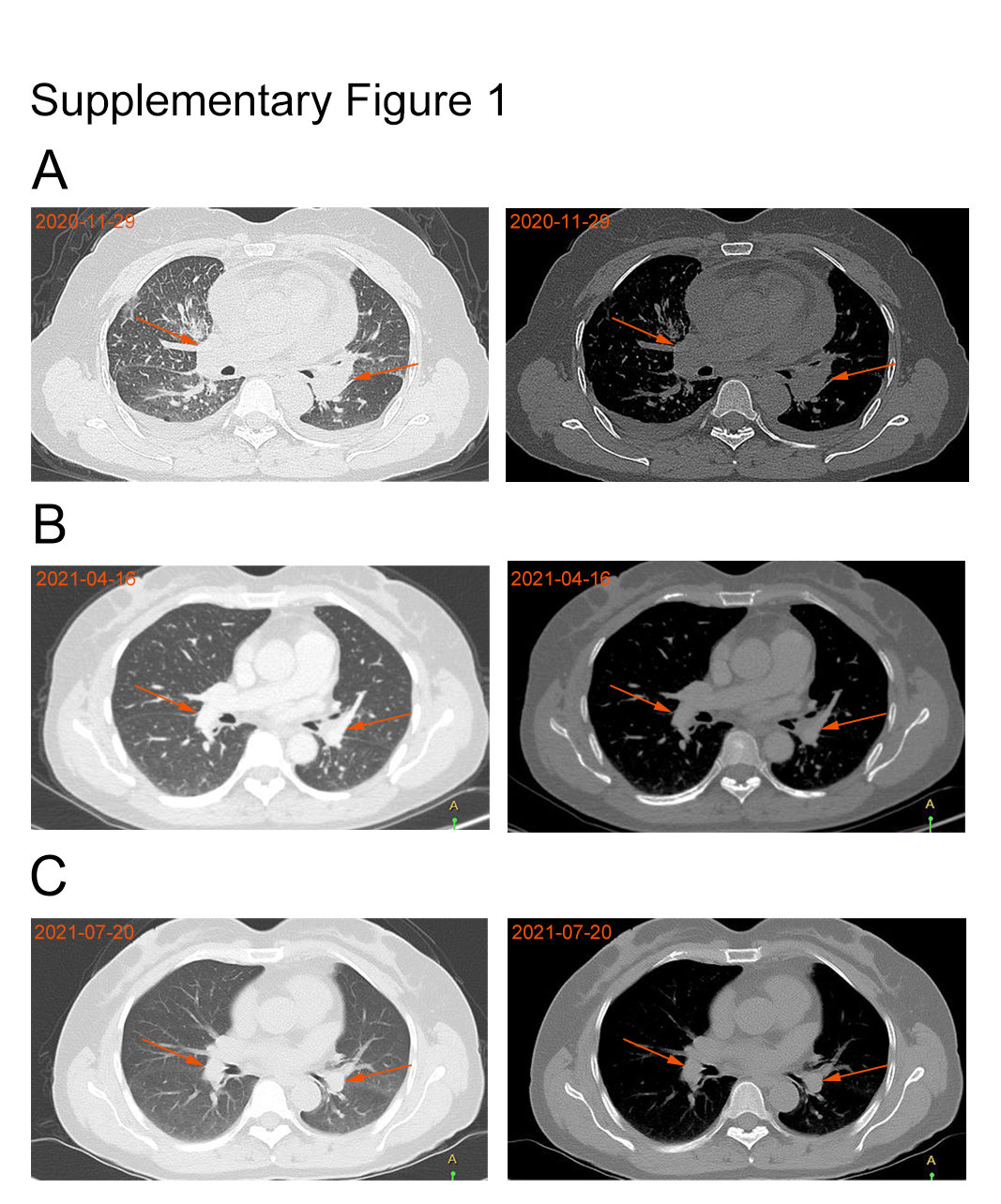

Supplement: Supplementary Figure 1 — Imaging results in the period between diagnosis and metastasis(red arrow: lung adenocarcinoma). (A) CT image before Camrelizumab+Alimta+Carboplatin. (B) CT image after Camrelizumab+Alimta+Carboplatin. (C) CT image after Camrelizumab+Alimta. [file Image_1.tif]

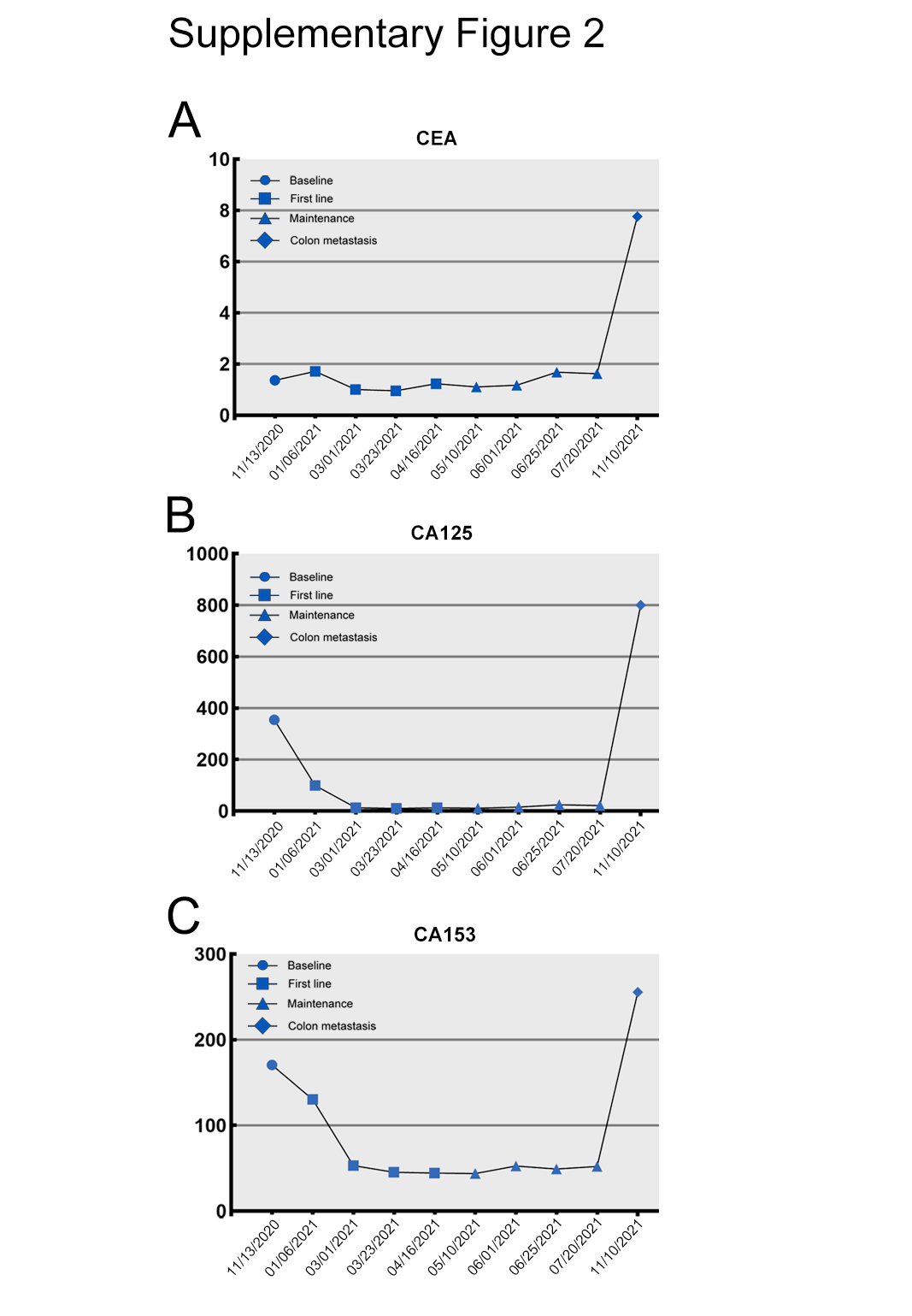

Supplement: Supplementary Figure 2 — Laboratory results in the period between diagnosis and metastasis. (A) Graph showing the dynamics of CEA tumor marker response during treatment. (B) Graph showing the dynamics of CA125 tumor marker response during treatment. (C) Graph showing the dynamics of CA153 tumor marker response during treatment. [file Image_2.tif]
